# Impact of sympathetic nervous system on immune evasion in high-grade serous ovarian cancer: a review

**DOI:** 10.3389/fonc.2025.1644895

**Published:** 2025-08-08

**Authors:** Ruiyuan Yu, Yumeng Li, Runze Jiang, Chunxiao Dang, Fengting Zhai

**Affiliations:** ^1^ Department of Gynecology, Shandong University of Traditional Chinese Medicine, Jinan, China; ^2^ Department of Gynecology, Shandong University of Traditional Chinese Medicine Affiliated Hospital, Jinan, China

**Keywords:** high-grade serous ovarian cancer, immune evasion, sympathetic nervous system, tumor microenvironment, immunotherapy

## Abstract

As the most frequent and aggressive subtype of ovarian cancer, high-grade serous ovarian cancer (HGSOC) often advances unnoticed due to its subtle early symptoms, which in turn leads to a significantly low five-year survival rate. The process of immune evasion, often achieved by constructing an immunosuppressive microenvironment through various pathways, stands as a critical feature of tumor biology. At the same time, emerging studies reveal a strong association between the sympathetic nervous system (SNS) and immune regulation in the tumor microenvironment (TME). In HGSOC, SNS activation releases neurotransmitters like norepinephrine, which affect immune cells, suppress their functions, weaken anti-tumor responses, and promote the recruitment and activation of immunosuppressive cells. By recruiting immune-suppressive cells, altering the extracellular matrix to construct physical barriers, and increasing pro-angiogenic signals, the SNS reshapes the tumor microenvironment in a way that hampers immunotherapy. Clinically, higher levels of SNS activation are linked to worse outcomes and therapeutic resistance in HGSOC. Additionally, preclinical studies demonstrate that targeting the SNS using β-adrenergic receptor inhibitors can improve immune activation and enhance treatment responses. Moving forward, research needs to further examine SNS mechanisms to support the development of advanced therapeutic strategies.

## 1 Introduction

High-grade serous ovarian cancer (HGSOC) accounts for the majority of malignant ovarian tumors and is widely recognized as the most aggressive subtype. Despite improvements in surgical techniques and chemotherapy regimens, its five-year survival rate remains low, largely due to its rapid progression and tendency to be diagnosed at advanced stages (1). Therefore, the aggressive and recurrent nature of HGSOC highlights an urgent demand for therapeutic strategies that specifically target its distinct biological mechanisms (2, 3).

As a defining feature of cancer progression, immune evasion permits malignant cells to bypass immune surveillance and resist immune-mediated clearance (4). In HGSOC, the mechanisms of immune evasion are particularly complex, involving the downregulation of antigen-presenting molecules, secretion of immunosuppressive factors, and recruitment of immunosuppressive cells (5–7). Together, these mechanisms shape an immunosuppressive tumor microenvironment (TME), which in turn facilitates unchecked tumor proliferation and metastatic spread.

In recent years, cancer neuroimmunology uncovers the complex interplay between the nervous system and immune responses within the tumor microenvironment (TME) (8, 9). Although the sympathetic nervous system (SNS) is mainly associated with the “fight or flight” mechanism, mounting evidence indicates that it communicates with both immune and tumor cells through neurotransmitters and corresponding receptors (10, 11). Consequently, the SNS modulates immune activity and, at the same time, promotes tumor development by reshaping the TME (12).

In HGSOC, SNS activation influences tumor immune responses through multiple mechanisms. For instance, neurotransmitters released by the SNS, such as norepinephrine, can directly affect immune cells, inhibiting their functions and weakening the anti-tumor immune response (13, 14). Moreover, when the SNS becomes activated, it contributes to immune escape by encouraging both the recruitment and functional activation of cells with immunosuppressive roles, such as Tregs and MDSCs (15).

**Figure 1:**
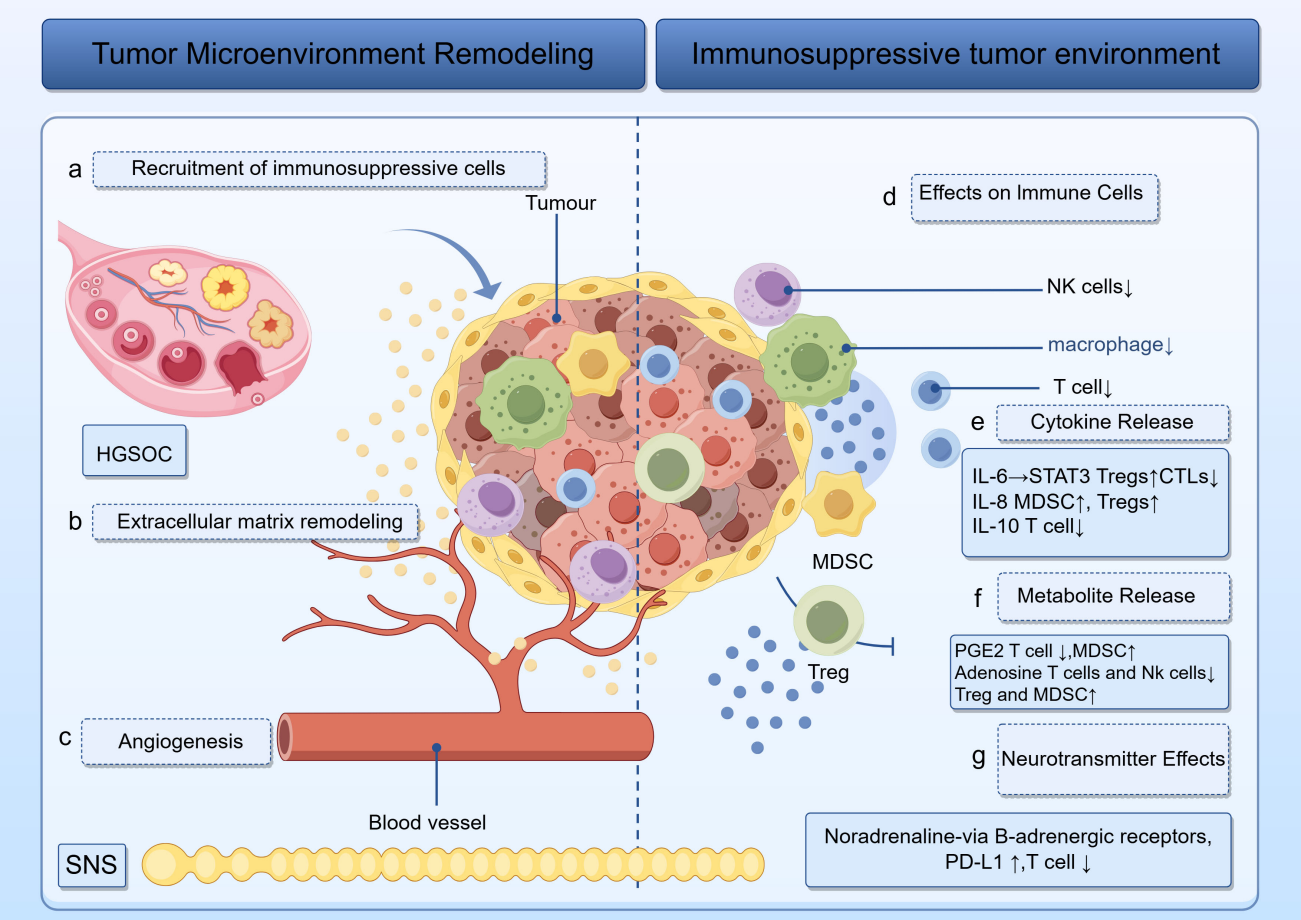
Illustration of how tumors sculpt a hostile microenvironment. Left: panels **(a–c)** track the recruitment of immunosuppressive cells, extracellular matrix remodeling and angiogenesis. Right: panels **(d–g)** trace the release of IL-6, IL-8, IL-10, PGE2, and noradrenaline, which act on T cells, NK cells, macrophages, MDSCs, and Tregs to enforce an immunosuppressive phenotype. The roles of HGSOC and the SNS are visually emphasized throughout.

Altogether, these observations build a valuable conceptual model for crafting new treatment paradigms. Modulating the interaction between the SNS and immune suppression could reactivate immune surveillance, promote tumor-specific immune responses, and lead to better clinical outcomes and life quality in HGSOC patients. Thus, future investigations should further define how the SNS regulates immunity and seek out more advanced therapeutic alternatives. The mechanistic diagram of this study is presented as **Figure 1**.

## 2 The SNS: basics and its role in tumor biology

The SNS is made up of a complex network of neurons that begin in the thoracolumbar area of the spinal cord and travel to peripheral organs through sympathetic ganglia. Its core neurotransmitters—norepinephrine and epinephrine—exert biological effects by binding to adrenergic receptors on a wide array of cell types. These receptors, part of the G protein-coupled receptor class, trigger diverse intracellular signaling pathways once activated (16).

In the context of HGSOC, tumor progression continuously initiates a prolonged stress reaction that activates the SNS. In addition, tumor-derived substances such as cytokines and growth factors activate afferent sensory nerves and spinal reflex pathways, resulting in enhanced sympathetic tone. Psychological distress commonly experienced by cancer patients further reinforces this loop, driving a sustained elevation in SNS activity (17).

Excessive activation of the sympathetic nervous system (SNS) not only undermines immune defenses but also profoundly affects tumor behavior. Through adrenergic signaling, the SNS promotes tumor cell proliferation. Moreover, it elevates vascular endothelial growth factor (VEGF) expression, thereby stimulating angiogenesis and improving the delivery of oxygen and nutrients to support tumor growth. Additionally, the SNS regulates cell adhesion molecules and extracellular matrix (ECM) elements, which aids in both local invasion and distant metastasis (18, 19).

These combined effects create a microenvironment that favors tumor growth and progression. Specifically, the enhanced proliferative signals allow tumor cells to divide and expand more rapidly (20). Enhanced angiogenesis not only delivers an abundant blood supply to the tumor but also promotes the spread of tumor cells through the circulatory system. Moreover, changes in the ECM further support the migration and invasion of cancer cells. In summary, the multifaceted impact of SNS activation in HGSOC accelerates disease progression, complicates treatment, and adversely affects patient prognosis.

## 3 Mechanisms of SNS-mediated immune evasion in HGSOC

### 3.1 Remodeling of the TME

By releasing neurotransmitters such as norepinephrine, the SNS draws immunosuppressive cells—including Tregs, MDSCs, and tumor-associated macrophages—into the ovarian cancer microenvironment, thereby contributing to immune evasion (21, 22). As they accumulate in the TME, these cells develop a powerful suppressive system that disrupts the roles of cytotoxic T cells and natural killer (NK) cells, and as a result, weakens the immune defense against the tumor (23, 24). Tregs, for example, produce factors like IL-10 and TGF-β, which in turn act to directly prevent the activation and proliferation of T lymphocytes. Furthermore, MDSCs secrete enzymes such as IDO and arginase, depleting essential nutrients like tryptophan, which leads to T-cell dysfunction (25).

In addition, through modulation of the ECM components, SNS signaling contributes to the formation of a physical barrier, which in turn restricts the entry of immune cells into the tumor site. Through the modulation of ECM components, SNS signaling helps establish a structural barrier that limits immune infiltration. When SNS responsiveness increases in HGSOC, it drives matrix metalloproteinases (MMPs) expression and ECM disorganization, resulting in fibrotic zones that obstruct immune cells and protect tumor cells, thus promoting immune evasion (26, 27).

Moreover, SNS signaling boosts the expression of vascular growth promoters, particularly VEGF, thus encouraging the formation of atypical and disorganized blood vessels. These irregular blood vessels are disorganized, leading to insufficient blood perfusion and hypoxia, which further exacerbates immune suppression. Additionally, the abnormal vascular network obstructs immune cell infiltration, further enhancing the immunosuppressive nature of the TME (28, 29).

These SNS-mediated microenvironmental remodeling mechanisms underscore the intricate crosstalk between the nervous and immune systems within the tumor microenvironment and profoundly influence therapeutic outcomes in ovarian cancer. For example, the limited efficacy of immune checkpoint inhibitors (ICIs) in ovarian cancer is often attributed to the highly immunosuppressive nature of the SNS-influenced TME, which impairs effective anti-tumor immune responses and hampers immunotherapeutic success. Targeting SNS signaling pathways may improve the TME and enhance the effectiveness of immunotherapy (30). For instance, combining β-blockers with ICIs could potentially boost anti-tumor immune responses by inhibiting SNS-mediated immune suppression.

### 3.2 Direct effects on immune cells

In HGSOC, the SNS exerts a direct influence on immune cell function by releasing neurotransmitters like norepinephrine, which suppresses the anti-tumor immune response (31, 32). Initially, SNS has a significant impact on innate immune cells. Norepinephrine binds to adrenergic receptors on NK cells, macrophages, and dendritic cells (DCs), inhibiting their functions. When SNS activation occurs, NK cells’ cytotoxic function is diminished, making it more difficult for them to identify and eliminate tumor cells. Macrophages also exhibit impaired phagocytosis, which hinders their ability to eliminate tumors. Similarly, DCs’ ability to present antigens is compromised, which hinders T cell activation and ultimately postpones adaptive immune responses. Together, these deficiencies weaken the innate immune system’s defenses against HGSOC, which aids in tumor immune evasion (33).

Similarly, adaptive immunity is also impacted by the SNS. It suppresses the tumor-killing potential of Cytotoxic T lymphocytes (CTLs), limits their growth, and lowers their production of interferon-γ (17).SNS suppresses CTL function through several mechanisms, including decreasing their proliferation, reducing the secretion of key cytokines like interferon-γ, and inhibiting their cytotoxic activity. Consequently, the immune system fails to establish a durable memory response against HGSOC cells, which permits the tumor to evade immune detection over time. This mechanism of immune escape plays a vital role in HGSOC progression and supports the rationale for designing novel therapeutic strategies.

### 3.3 Induction of immunomodulatory factors

SNS activation triggers the release of multiple immune-regulating factors into the TME, which together create an immunosuppressive milieu that promotes tumor growth and metastasis. Among these factors, cytokines like interleukin-6 (IL-6), interleukin-8 (IL-8), and interleukin-10 (IL-10) are widely released after SNS stimulation (34). IL-6 is a multifunctional cytokine that activates the STAT3 signaling pathway, which promotes the growth of Tregs while inhibiting the function of cytotoxic T cells; IL-8 primarily improves immune suppression by attracting MDSCs and Tregs (35); IL-10 significantly lowers the activation and proliferation of T cells by inhibiting antigen-presenting cells like dendritic cells, which contributes to immune suppression (36).

Importantly, these cytokines may serve as potential biomarkers for clinical application. Measuring circulating levels of IL-6, IL-8, and IL-10 in patients with high-grade serous ovarian cancer (HGSOC) could provide insights into the degree of SNS-driven immunosuppression, which may correlate with disease progression or therapeutic resistance. Clinically, these biomarkers could aid in stratifying patients according to immune risk, identifying those who might benefit from anti-inflammatory or sympathetic blockade therapies. Additionally, incorporating cytokine profiles into treatment planning may help predict responses to immunotherapy and guide the use of immune-modulating agents in personalized treatment strategies.

Beyond its direct effects on immune cell function, activation of the SNS also influences the tumor microenvironment through the release of metabolic mediators such as adenosine and prostaglandins. These mediators, regulated in part by adrenergic signaling, represent a critical link between neural input and immune modulation. Notably, PGE2 plays a key immunosuppressive role via multiple pathways. PGE2 attenuates anti-tumor immunity by directly inhibiting T cell proliferation and cytokine secretion, while concurrently enhancing the expansion and immunosuppressive activity of myeloid-derived suppressor cells (MDSCs). This highlights the complex neuro-immune-metabolic interplay orchestrated by the SNS within the tumor microenvironment (37), weakening the anti-tumor immune response. Adenosine suppresses the activity of T cells and NK cells and interacts with immune cells by binding to A2A receptors, which modifies their function (38). Adenosine also increases the number of Treg cells and attracts MDSCs, which strengthens immune suppression.

SNS activation also exerts a direct effect on immune cells through the release of neurotransmitters like norepinephrine. Norepinephrine activates intracellular signaling pathways in immune cells through β-adrenergic receptors, leading to an increase in the expression of immunosuppressive factors such as PD-L1, which inhibits T-cell function.

## 4 Clinical evidence and therapeutic implications

According to clinical studies, biomarkers of SNS activation, including increased plasma norepinephrine levels, are strongly linked to poor prognosis, therapy resistance, and immune suppression in HGSOC patients (39, 40). More specifically, patients with elevated sympathetic nervous activity often show lower survival rates and higher recurrence rates, emphasizing the clinical importance of targeting the SNS in these individuals (41). Such observations provide a basis for designing innovative treatments and underscore the necessity to explore the SNS involvement in HGSOC more thoroughly.

*In vitro* and *in vivo* research demonstrates that regulating the SNS with β-adrenergic blockers and other drugs can significantly influence immune evasion and tumor progression in HGSOC models (42). Propranolol, as a β-blocker, suppresses tumor angiogenesis, enhances the penetration of T cells within tumors, and lowers the quantity of MDSCs (43). As a result, these changes boost the anti-tumor immune response and increase the effectiveness of ICIs, such as anti-CTLA4 antibodies (44). Moreover, these studies offer robust evidence that supports translating preclinical results into clinical practice and emphasize the potential of SNS-targeted therapies.

Drugs targeting adrenergic receptors, such as β-adrenergic receptor antagonists and α-adrenergic receptor antagonists, show great potential in enhancing the effectiveness of immunotherapy (45). These drugs work by disrupting the SNS-immune evasion axis, thereby restoring immune cell function and improving anti-tumor immunity. For instance, propranolol promotes a change in the tumor microenvironment toward a pro-inflammatory condition, thereby potentially enhancing the effects of ICI treatments (46). Research demonstrates that combining propranolol with anti-CTLA4 therapy significantly improves treatment outcomes, reduces tumor growth rates, and increases overall survival.

The combination of SNS-targeted therapy with ICIs, chemotherapy, or targeted therapies represents a promising therapeutic approach. Preclinical research demonstrates that these combined methods have synergistic effects, allowing for the targeting of multiple pathways at once to overcome resistance and improve clinical outcomes (47). In some cases, combination therapies even lead to the complete elimination of tumors and the achievement of long-lasting anti-tumor responses.

Additionally, changes in lifestyle, such as stress management, regular physical activity, and psychological support, can regulate the SNS and enhance immune function in HGSOC patients. These non-drug interventions provide an important supplement to traditional treatments, addressing the broader needs of cancer patients. By integrating drug therapies with lifestyle adjustments, it is possible to more effectively modulate the SNS and ultimately improve prognosis and quality of life for HGSOC patients

## 5 Future directions and challenges

Although significant progress has been made in understanding the interaction between the SNS and immune evasion in HGSOC, many unresolved issues remain. These include understanding the heterogeneity of SNS responses among patients, identifying specific molecular pathways, and determining the long-term impact of SNS regulation on immune function and tumor biology.

Emerging technologies, such as single-cell sequencing, *in vivo* imaging, and organoid models, provide unprecedented opportunities to gain deeper insights into the SNS-immune-tumor interactions. Single-cell sequencing enables precise measurements of the genome and transcriptome of individual cells, which helps identify new mutations in cancer cells and explore cellular heterogeneity within the TME (48, 49). *In vivo* imaging allows real-time observation of cellular behavior and interactions in the TME, offering a powerful tool to study the dynamic role of SNS signaling in tumor progression. Organoid models, on the other hand, simulate the microenvironment of real organs, providing a new platform for drug screening, studying resistance mechanisms, and investigating the role of SNS signaling in tumor immune evasion (50).

Designing clinical trials to assess SNS-targeted therapies in HGSOC requires careful consideration of patient selection, biomarker identification, and combination therapy strategies. Future trials should include comprehensive assessments of immune function and sympathetic nervous activity to better understand the clinical impact of these interventions. For instance, by designing umbrella, basket, or platform trials, patients can be stratified based on biomarkers, allowing more effective evaluation of SNS-targeted therapy efficacy across different patient groups. This approach not only accelerates clinical trial progress but also provides essential support for personalized medicine.

## 6 Conclusion

HGSOC represents the most common and aggressive subtype of ovarian cancer, posing a major challenge in gynecologic oncology due to its high recurrence rate and poor survival outcomes (51–53). Although surgical intervention and chemotherapy partially improve patient prognosis, immune evasion and the complexity of the TME remain key factors influencing treatment efficacy. In recent years, the role of the SNS in tumor immune evasion has gained increasing attention (54). Specifically, in HGSOC, SNS shapes an immunosuppressive TME through multiple mechanisms, thereby accelerating tumor progression. This study integrates existing research to explore the mechanisms of SNS in HGSOC and its therapeutic potential.

First, by impacting immune cells, SNS reduces the immune response against tumors. SNS-released neurotransmitters, like norepinephrine, attach to immune cells’ adrenergic receptors and prevent NK cells from being cytotoxic, macrophages from being phagocytic, and DCs from presenting antigens. Consequently, the ability of the innate immune system to identify and eliminate tumor cells declines (55). Moreover, SNS suppresses the proliferation of CTLs and downregulates cytokines such as interferon-gamma, which in turn compromises the function of the adaptive immune system (56). Consequently, the simultaneous suppression of immune responses enables tumor cells to avoid sustained immune detection, thus driving disease advancement.

Secondly, SNS further exacerbates immunosuppression by inducing the release of immunoregulatory factors. Upon SNS activation, cytokine levels—including IL-6, IL-8, and IL-10—are significantly elevated in the TME. These cytokines not only suppress immune cell activity but also promote the expansion of Tregs, forming an immunosuppressive network (57, 58). Additionally, SNS-induced metabolic byproducts such as prostaglandins and adenosine further inhibit T-cell proliferation and immune cell activation, thus weakening anti-tumor immunity.

In addition, the SNS accelerates tumor development through remodeling of the tumor microenvironment. It attracts various immunosuppressive populations, such as Tregs, MDSCs, and tumor-associated macrophages, which collectively create an immunosuppressive landscape (59). Concurrently, it alters ECM composition, forming a physical barrier that restricts the access of immune cells to the tumor (60). Furthermore, SNS enhances tumor angiogenesis by upregulating VEGF, supporting tumor growth and metastasis.

Clinical studies reveal a strong correlation between SNS overactivation and poor prognosis in HGSOC patients. For instance, elevated norepinephrine levels in plasma are often associated with lower survival rates and higher recurrence rates. These findings provide a theoretical basis for SNS-targeted therapy. *In vivo* and *in vitro* studies demonstrate that beta-adrenergic blockers significantly enhance anti-tumor immunity and inhibit tumor growth (61). Additionally, combining SNS-targeted therapy with ICIs, chemotherapy, or targeted therapies may synergistically overcome treatment resistance, ultimately improving patient outcomes.

However, several unresolved questions remain regarding the role of SNS in HGSOC. For example, does patient heterogeneity influence SNS response? Which molecular pathways play pivotal roles in the SNS-mediated immune evasion axis? Novel approaches such as single-cell genomics, dynamic imaging systems, and patient-derived organoid platforms should be used to decipher the complex interactions among SNS, immunity, and tumor progression. Moreover, careful trial design that incorporates validated biomarkers, combinatorial treatment strategies, and patient selection algorithms is necessary to optimize SNS-based clinical interventions. When taken as a whole, this research paradigm has the potential to revolutionize our understanding of HGSOC carcinogenesis. In the future, intervention in the immune microenvironment of ovarian cancer will enter an era of “precise regulation of specific immune cell subsets” and will be highly dependent on the deep integration of multidisciplinary technologies (62–64).

In summary, investigating the role of the SNS in HGSOC offers novel insights for advancing tumor immunotherapy. Targeting the SNS immune evasion axis, especially in combination with current treatment modalities, holds considerable potential to improve patient outcomes. Future research leveraging tools such as single-cell transcriptomics, patient-derived xenograft models, and stress-response profiling is essential to unravel SNS heterogeneity and downstream molecular pathways, ultimately guiding the development of more effective and personalized therapeutic strategies (65, 66).
